# Artificial Intelligence for Early Detection of Preeclampsia and Gestational Diabetes Mellitus: A Systematic Review of Diagnostic Performance

**DOI:** 10.7759/cureus.92585

**Published:** 2025-09-17

**Authors:** Sahar Altayeb Alfaki Ahmed, Mohammedelfateh Adam, Hanady ME M Osman, Naif Hadi Fahad Alqahtani, Abeer Ebaid Mahdi Gabreldaar, Mona Sidahmed Hassan Abdalla, Ryan Osman Alhessen Saidahmed

**Affiliations:** 1 Obstetrics and Gynecology, King Khaled Majmaah Hospital, Riyadh, SAU; 2 Obstetrics and Gynecology, Cork University Maternity Hospital, Cork, IRL; 3 Quality Improvement and Patient Safety, Najran Armed Forces Hospital, Ministry of Defense Health Services, Najran, SAU; 4 Pharmacy, Najran Armed Forces Hospital, Ministry of Defense Health Services, Najran, SAU; 5 Obstetrics and Gynecology, National Guard Health Affairs-Specialized Hospital for Women’s Health, Riyadh, SAU; 6 Obstetrics and Gynecology, Maternity and Children Hospital, Buraydah, SAU; 7 Obstetrics and Gynecology, Sabt Al Alayah General Hospital, Sabt Al Alayah, SAU

**Keywords:** artificial intelligence, diagnostic performance, early diagnosis, gestational diabetes, machine learning, predictive models, preeclampsia, systematic review

## Abstract

Preeclampsia (PE) and gestational diabetes mellitus (GDM) are major contributors to maternal and neonatal morbidity and mortality. Early detection is critical, yet current approaches, such as clinical risk scores for PE and glucose challenge/oral glucose tolerance test (OGTT) screening for GDM, often show limited sensitivity and variable predictive accuracy. Artificial intelligence (AI) and machine learning (ML) offer promising avenues for enhancing early prediction and diagnosis. This systematic review, conducted in accordance with the Preferred Reporting Items for Systematic reviews and Meta-Analyses (PRISMA) guidelines, synthesized evidence from five databases (PubMed, Scopus, Embase, IEEE Xplore, ACM Digital Library) covering January 2020-July 2025. Eligible studies included both model development and validation efforts in pregnant populations. Data were extracted on study characteristics, AI model types, and diagnostic performance metrics. Risk of bias was assessed using the Prediction model Risk of Bias Assessment Tool (PROBAST). Nine studies met the inclusion criteria, reflecting strict eligibility requirements and limited high-quality research in this area. AI models frequently achieved strong performance, with area under the curve (AUC) values often >0.85. For PE, a neural network model externally validated in Spain achieved AUCs of 0.920 and 0.913 for early and preterm PE, with sensitivity up to 84%. For GDM, an XGBoost model achieved an AUC of 0.946 with an accuracy of 87.5%, while a Random Forest model reached a sensitivity of 75-85% and a specificity of 88-91%. Ensemble methods generally outperformed logistic regression. Seven studies were judged low risk of bias, while two were high risk, particularly in participant selection and analysis domains. Several models also demonstrated good calibration and positive net benefit on decision curve analysis, comparable to established clinical tools. AI models show substantial potential for early detection of PE and GDM, though heterogeneity and limited external validation remain barriers. Future research should prioritize multicenter, prospective validation, standardized reporting, and attention to equity and generalizability to ensure safe and effective translation into clinical practice.

## Introduction and background

Preeclampsia (PE) and gestational diabetes mellitus (GDM) are two of the most common and clinically significant pregnancy complications, posing substantial risks to both maternal and neonatal health [1]. PE, characterized by hypertension and organ dysfunction after 20 weeks of gestation, affects approximately 2-8% of pregnancies worldwide and is a leading cause of maternal morbidity, mortality, preterm birth, and intrauterine growth restriction [2]. Similarly, GDM, defined as glucose intolerance with onset or first recognition during pregnancy, shows wide variability in prevalence depending on diagnostic criteria and geography, ranging from about 1% to 28% globally, and is associated with adverse perinatal outcomes as well as long-term risks of type 2 diabetes and cardiovascular disease in both mother and child [3]. Early detection and timely intervention for these conditions are critical for improving clinical outcomes, reducing healthcare burdens, and preventing long-term complications.

Traditional diagnostic strategies for PE and GDM largely rely on clinical assessment, biochemical markers, and standard laboratory tests [4]. For PE, established risk models such as those endorsed by the National Institute for Health and Care Excellence (NICE), American College of Obstetricians and Gynecologists (ACOG), and the Fetal Medicine Foundation (FMF) algorithm achieve only moderate predictive accuracy, with typical area under the curve (AUC) values around 0.70-0.75. For GDM, the oral glucose tolerance test (OGTT), the current diagnostic standard, is inconvenient, performed relatively late in pregnancy (24-28 weeks), and unsuitable for early prediction. These limitations, compounded by variability in population characteristics, clinical practice guidelines, and resource availability, particularly in low- and middle-income countries, highlight the urgent need for more robust and accessible diagnostic tools [5].

Recent advances in artificial intelligence (AI) and machine learning (ML) have opened new possibilities for enhancing early disease detection in obstetrics [6]. AI algorithms can integrate complex, high-dimensional data such as maternal demographics, clinical histories, laboratory parameters, imaging, and even omics to generate predictive models with superior accuracy [7]. Applications are expanding beyond PE and GDM, with promising results already reported in fetal growth assessment, preterm birth prediction, and noninvasive monitoring. Within this context, ML methods (e.g., logistic regression, support vector machines, random forests) and deep learning techniques (e.g., neural networks) are increasingly applied, with the latter showing particular promise for uncovering subtle, nonlinear patterns not captured by traditional approaches [8]. In PE and GDM, such models hold promise for enabling earlier diagnosis, personalized risk stratification, and better allocation of preventive interventions, ultimately contributing to safer pregnancies and healthier outcomes [9].

Despite the growing body of literature, the diagnostic performance of AI models remains heterogeneous, reflecting variation in input features, algorithms, validation strategies, and outcome measures. Standardized reporting guidelines such as the Transparent Reporting of a multivariable prediction model for Individual Prognosis Or Diagnosis (TRIPOD-AI) and Consolidated Standards of Reporting Trials-Artificial Intelligence (CONSORT-AI) are emerging but inconsistently applied, contributing to heterogeneity. Furthermore, external validation and clinical utility assessment, beyond statistical performance alone, are critical for determining real-world applicability. A systematic synthesis of the available evidence is therefore essential to evaluate the accuracy, strengths, and limitations of AI applications in this domain.

The present systematic review focuses on studies published between 2020 and 2025 and aims to comprehensively assess the diagnostic performance of AI models developed for the early detection of PE and GDM. By examining sensitivity, specificity, accuracy, AUC, and other performance indicators, and by assessing study quality with the Prediction model Risk of Bias Assessment Tool (PROBAST), this review seeks to determine the potential role of AI in advancing obstetric care and improving maternal-fetal health outcomes.

## Review

Methodology

Eligibility Criteria

The eligibility of studies was defined a priori based on the population, intervention, comparator, outcomes, and study (PICOS) design framework. Studies were included if they evaluated the diagnostic performance of AI or ML models in the early detection of PE or GDM. Studies published between January 2020 and July 2025 were included in order to ensure that only recent studies were included. The inclusion and exclusion criteria are summarized in Table 1.

**Table 1:** Eligibility criteria for inclusion and exclusion AI: artificial intelligence; ML: machine learning; GDM: gestational diabetes mellitus

Criterion	Inclusion	Exclusion
Population	Pregnant women undergoing screening or diagnosis for preeclampsia or GDM	Nonpregnant women, animal studies, in vitro studies
Intervention	AI/ML models developed or validated for early detection or prediction of preeclampsia and/or GDM	Studies not using AI/ML approaches (e.g., only statistical regression without ML techniques)
Comparator	Standard diagnostic approaches (clinical, biochemical, imaging, or established guidelines) or no comparator	Studies with no clear diagnostic comparator or reference standard
Outcomes	Diagnostic performance measures such as sensitivity, specificity, accuracy, area under the curve (AUC), predictive values, or related metrics	Studies reporting only nondiagnostic outcomes (e.g., treatment efficacy, prognosis, cost analysis)
Study design	Original research studies, including prospective, retrospective, case-control, and cohort studies	Reviews, editorials, commentaries, conference abstracts without full text, case reports, letters
Language	English	Non-English publications
Publication type	Peer-reviewed journal articles	Grey literature, dissertations, preprints without peer review

Information Sources

A comprehensive literature search was performed across five major electronic databases: PubMed, Scopus, Embase, IEEE Xplore, and ACM Digital Library. These databases were selected to ensure wide coverage of both biomedical and computer science literature. The search covered the period from January 2020 to July 2025, and search alerts were set to capture studies published close to the cut-off date. Additional manual searches were performed by screening the reference lists of relevant studies to identify potential articles missed in the electronic search.

Search Strategy

The search strategy was designed in consultation with domain experts and tailored to each database using controlled vocabulary (e.g., medical subject headings (MeSH) terms in PubMed) and keywords related to preeclampsia, gestational diabetes mellitus, artificial intelligence, and relevant concepts such as “prediction,” “risk stratification,” and “screening,” in addition to “diagnosis.” Boolean operators, truncation, and database-specific filters were applied as appropriate. The search strategy was peer-reviewed using the Peer Review of Electronic Search Strategies (PRESS) checklist to ensure completeness and accuracy. An example of the PubMed search strategy is shown in Table 2.

**Table 2:** Search strategy example (PubMed)

Search concept	Search terms
Preeclampsia	“Preeclampsia” OR “Pregnancy-Induced Hypertension” OR “Hypertensive Disorders of Pregnancy”
Gestational diabetes mellitus	“Gestational Diabetes” OR “GDM”
Artificial intelligence	“Artificial Intelligence” OR “Machine Learning” OR “Deep Learning” OR “Neural Network” OR “Support Vector Machine” OR “Random Forest”
Combined search	(Preeclampsia OR Gestational Diabetes OR GDM) AND (“Artificial Intelligence” OR “Machine Learning” OR “Deep Learning”)

Equivalent strategies were adapted for Scopus, Embase, IEEE Xplore, and ACM Digital Library to ensure comprehensive coverage. The search strings for these databases are supplemented in the appendices section.

Selection Process

**Figure 1:**
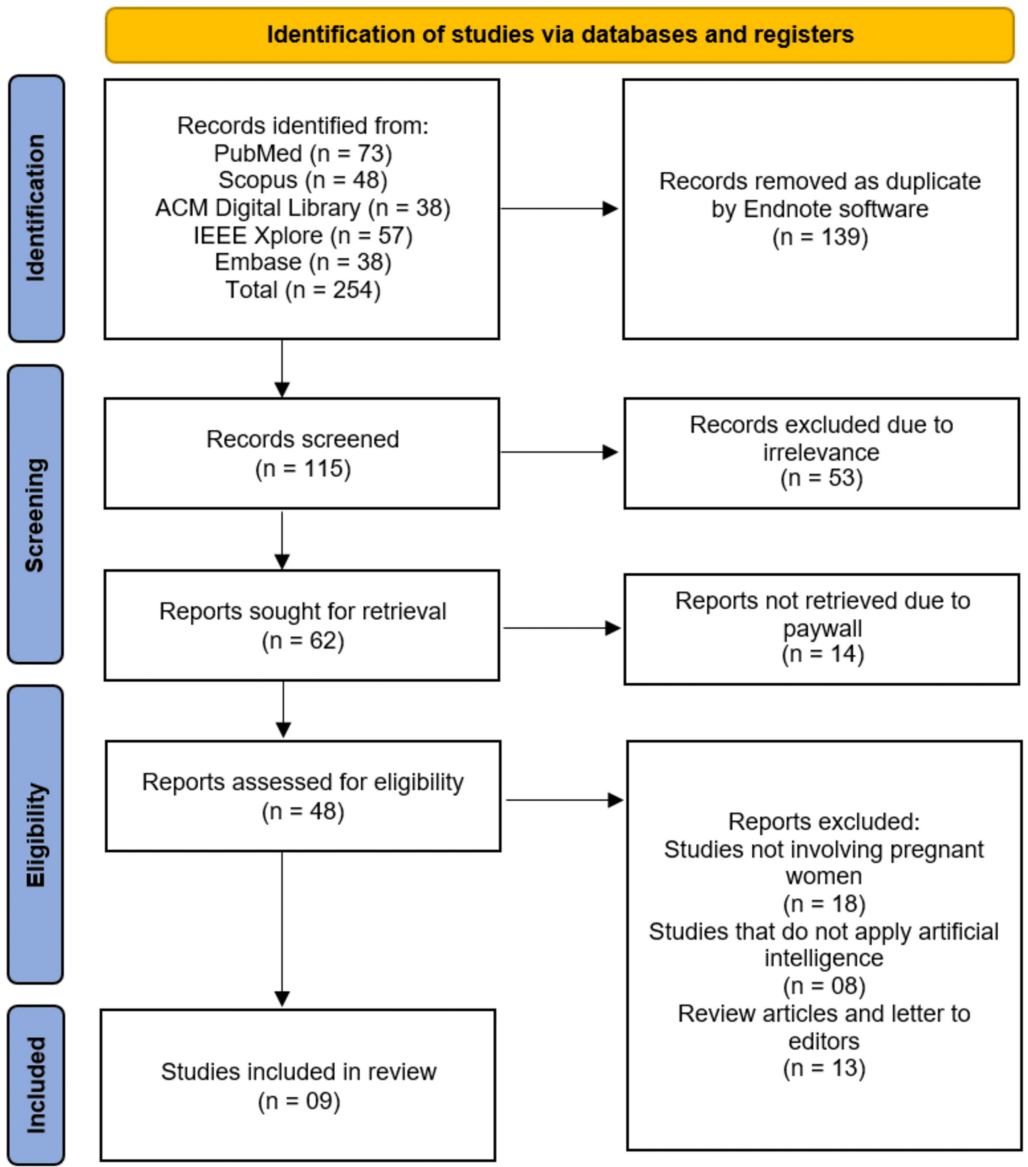
Studies selection process

All retrieved records were exported into EndNote (version X9) for management. Duplicate references were identified and removed both automatically and manually. Titles and abstracts were independently screened by two reviewers, who were blinded to author and journal information, to assess relevance based on eligibility criteria. Potentially eligible studies were retrieved in full text and independently assessed by the same reviewers. Discrepancies were resolved through discussion, and if necessary, a third reviewer was consulted. A Preferred Reporting Items for Systematic reviews and Meta-Analyses (PRISMA) flow diagram was generated to illustrate the study selection process (Figure 1).

Data Collection Process

Data extraction was carried out independently by two reviewers using a predesigned extraction form, which was piloted on a subset of studies before full extraction. The form captured information on study characteristics, population demographics, AI/ML model type, input features, validation method, reference standard, diagnostic performance metrics (sensitivity, specificity, accuracy, AUC, predictive values), and key conclusions. Any disagreements were resolved by consensus. Corresponding authors were contacted when data were missing or unclear.

Data Items

The following key data items were extracted: (1) study identifiers (author, year, country); (2) study design and population characteristics; (3) type of AI/ML model and training dataset; (4) input features used; (5) comparator or reference standard; (6) diagnostic performance indicators (sensitivity, specificity, accuracy, AUC, and other reported metrics); (7) calibration measures, such as calibration plots, Hosmer-Lemeshow test, and Brier score; and (8) decision curve analysis results, when reported, to assess model utility.

Study Risk of Bias Assessment

The PROBAST was used to assess the methodological quality and risk of bias of included studies [10]. The tool evaluates four domains: participants, predictors, outcomes, and analysis. Assessments were performed at the study level, as most studies reported a single primary model, making study-level evaluation most appropriate. Two reviewers independently performed the assessment, and disagreements were resolved by discussion or consultation with a third reviewer. Inter-rater agreement (e.g., kappa statistic) was not calculated because consensus discussion was used to resolve all discrepancies, ensuring accuracy and consistency in the final assessments.

Effect Measures

The primary effect measures considered were diagnostic accuracy indicators, including sensitivity, specificity, accuracy, AUC (95% CI), positive predictive value (PPV), and negative predictive value (NPV), where available. For AI studies, calibration measures (e.g., calibration plots, Hosmer-Lemeshow test, Brier score) and decision-curve analysis results were also extracted to assess model performance and clinical utility.

Synthesis Methods

Heterogeneity was assessed across study design, AI model type, input features, validation techniques, and outcome measures. Due to substantial heterogeneity, a meta-analysis was not performed, as pooling results across such diverse methodologies could introduce bias and misleading conclusions. Instead, findings were synthesized qualitatively and presented in tabular and narrative form. Studies were grouped thematically by disease (PE vs. gestational diabetes), AI model type (traditional ML vs. deep learning), and validation type (internal vs. external) to highlight trends, methodological differences, and diagnostic performance across studies.

Results

Study Selection Process

The study selection process is detailed in the PRISMA flow diagram (Figure 1). A total of 254 records were identified through systematic searches of the electronic databases: PubMed (n = 73), Scopus (n = 48), ACM Digital Library (n = 38), IEEE Xplore (n = 57), and Embase (n = 38). After removal of 139 duplicates by EndNote software, 115 unique records remained for title and abstract screening. Following this screening, 53 records were excluded due to irrelevance to the review’s primary focus. The full texts of the remaining 62 reports were sought for retrieval; however, 14 articles could not be accessed due to paywall restrictions despite attempts to obtain them through institutional access and interlibrary requests, which may introduce availability bias. Of the 48 reports that were successfully retrieved and assessed for eligibility, 39 were excluded for the following reasons: studies not involving pregnant women (n = 18), studies that did not apply AI or ML (n = 8), and review articles or letters to the editor (n = 13). Consequently, a total of nine studies [11-19] met all predefined inclusion criteria and were included in the qualitative synthesis, representing ~3.5% of the initially identified records, highlighting the stringency of the selection process.

Study Characteristics

A total of nine studies were included in this systematic review, the characteristics of which are summarized in Table 3 [11-19]. The studies were published between 2020 and 2025, with a notable concentration in recent years. Geographically, the majority of studies were conducted in China (n = 6) [13-16, 18, 19], with single studies from Spain (with a model developed in the UK) [12], the United States [17], and a large multicountry consortium spanning the Americas, sub-Saharan Africa, South Asia, Europe, and Oceania [11].

**Table 3:** Characteristics of included studies AI: artificial intelligence; ML: machine learning; PIERS-ML: preeclampsia integrated estimate of risk-machine learning model; fullPIERS: full preeclampsia integrated estimate of risk model (logistic regression); GA: gestational age; PE: preeclampsia; GDM: gestational diabetes mellitus; MAP: mean arterial pressure; UtA-PI: Uterine Artery Pulsatility Index; PlGF: placental growth factor; PAPP-A: pregnancy-associated plasma protein A; FMF: Fetal Medicine Foundation; DR: detection rate; SPR: screen-positive rate; XGBoost: extreme gradient boosting; BMI: body mass index; Hb: hemoglobin; NR: not reported; DNN: deep neural network; LR: logistic regression; SVM: support vector machine; DT: decision tree; RF: Random Forest; KPNC: Kaiser Permanente Northern California; CART: classification and regression tree; LASSO: least absolute shrinkage and selection operator; EHR: electronic health record; t-SNE: t-distributed stochastic neighbor embedding

Author, year	Country	Study design	Sample size (n)	Population (pregnant women characteristics)	Condition targeted	AI/ML model used	Data source	Reference standard
Yang et al. (2025) [11]	Multicountry (Americas, sub-Saharan Africa, South Asia, Europe, Oceania)	Prospective multicountry cohort; consecutive (daily) prediction performance evaluation over 2 weeks post-admission	8,843	Pregnant women admitted with preeclampsia (median age 31 years; 32% White, 30% Black, 26% Asian; median GA 35.79 weeks; admissions 2003-2016)	Preeclampsia-risk of adverse maternal outcomes during/after admission	PIERS-ML and fullPIERS (logistic regression)	Clinical data from maternity unit admissions across participating regions (2003-2016)	Composite adverse maternal outcome per PIERS definition: death, end-organ complications, or uteroplacental dysfunction, assessed daily within a 2-week post-admission window
Gil et al. (2024) [12]	Spain (model originally derived/tested in the UK; externally applied in Spain)	External validation/secondary analysis of a first-trimester PE screening cohort (PREVAL)	10,110	Singleton pregnancies undergoing first-trimester screening; maternal risk factors and biomarkers collected (MAP, UtA-PI, PlGF; PAPP-A assessed)	Preeclampsia (early <34 w, preterm <37 w, all PE)	Fully connected neural network (machine-learning model)	First-trimester screening dataset from the PREVAL study (Spain) including maternal history + biomarkers (MAP, UtA-PI, PlGF ± PAPP-A)	Performance compared against the FMF competing-risks model for PE screening (AUC and DR at 10% SPR); adjustment made for PlGF analyzer differences
Hu et al. (2023) [13]	China	Case-control study	925 (training: 735; testing: 190)	Pregnant women recruited Aug 2019-Nov 2019 (training) and Aug 2020 (testing)	GDM	Extreme gradient boosting (XGBoost); logistic regression	Clinical and demographic data (33 variables, reduced to 20 predictors in ML model)	Diagnosis of GDM
Li et al. (2023) [14]	China	Retrospective study	673 (182 GDM patients, 491 controls)	Pregnant women attending Xi’an International Medical Center Hospital (2018-2021); GDM cases and normal parturients	GDM	Logistic regression-based nomogram model	Hospital medical records (clinical and biochemical data)	Diagnostic criteria for GDM
Lin and Fang (2023) [15]	China	Retrospective cohort study	406	Pregnant women undergoing routine prenatal examination at Fujian Maternity and Child Health Hospital (April 2020-December 2022)	GDM	Logistic regression, Random Forest	Clinical and laboratory data (serum ferritin, fasting blood glucose, BMI, Hb, triglycerides, medical/family history)	Diagnosis of GDM (based on clinical criteria during pregnancy)
Liu et al. (2022) [16]	China	Retrospective study (medical records review)	NR	Pregnant women in early pregnancy undergoing prenatal screening	PE	DNN, LR, SVM, DT, RF	Retrospective medical records	Clinical diagnosis of preeclampsia
Liao et al. (2022) [17]	USA (Kaiser Permanente Northern California)	Population-based cohort (retrospective cohort design); discovery (2007-2016) and temporal/future validation (2017)	30,474 pregnancies with GDM	Pregnant women diagnosed with GDM who delivered at KPNC between 2007 and 2017. (No further demographic details reported in the abstract.)	Prediction of GDM treatment modality	Super Learner ensemble (CART, LASSO, Random Forest, XGBoost)	EHR from Kaiser Permanente Northern California; predictors extracted at multiple timepoints	Observed clinical treatment recorded in EHR-i.e., actual treatment modality as recorded in medical records
Kang et al. (2021) [18]	China (Shanghai)	Prospective cohort study	413	Pregnant women (gestational weeks 12-16 at enrollment, followed to 24-26 weeks)	GDM	Nomogram (based on multivariate logistic regression, validated with fivefold cross-validation)	Clinical and biochemical data (blood indexes, BMI, age, etc.) from Shanghai General Hospital	Diagnosis of GDM during follow-up
Guo et al. (2020) [19]	China (Shanghai)	Retrospective cohort study for model development (2015) with a prospective cohort validation (2016)	Development: 3,956; Validation: 6,572	Urban antenatal women who attended their first antenatal visit. Predictors used: age, prepregnancy BMI, first-trimester fasting plasma glucose, family history of diabetes	GDM	Multiple logistic regression → nomogram (prediction model). t-SNE used to refine/visualize separation between GDM and non-GDM. Decision curve analysis reported	Hospital antenatal clinical records from the institution in Shanghai (first antenatal visit data, first-trimester measures)	Clinical diagnosis of preeclampsia

The reviewed studies focused on two primary conditions: PE (n = 3) [11,12,16] and GDM (n = 6) [13-15, 17-19]. Sample sizes varied considerably, ranging from 406 [15] to 30,474 [17] participants. Study designs were predominantly retrospective cohorts (n = 5) [14-17,19], with the remainder being prospective cohorts (n = 3) [11,12,18] and one case-control study [13]. The populations consisted entirely of pregnant women, with studies either focusing on general obstetric populations undergoing screening or specific cohorts diagnosed with the target condition.

A diverse array of ML and statistical models was employed. For prediction, logistic regression-based nomograms were common for GDM [14,15,18,19]. More advanced ML algorithms included XGBoost [13,17], Random Forest [15,17], neural networks (a fully connected network [12] and a DNN [16]), and ensemble methods like the Super Learner [17]. The study by Yang et al. utilized the PIERS-ML and fullPIERS models for risk stratification in PE [11]. Data sources were primarily clinical and demographic variables extracted from hospital medical records, electronic health records (EHRs), or dedicated prenatal screening study datasets.

Diagnostic Performance of AI Models

The diagnostic performance metrics of the included AI models are detailed in Table 4. The AUC was the most consistently reported metric, with values indicating a range of performance from moderate to excellent.

**Table 4:** Diagnostic performance of AI models PIERS-ML: preeclampsia integrated estimate of risk-machine learning model; fullPIERS: full preeclampsia integrated estimate of risk model (logistic regression); NR: not reported; AUC: area under the curve; AUC-PRC: area under the precision-recall curve; PE: preeclampsia; GDM: gestational diabetes mellitus; MAP: mean arterial pressure; UtA-PI: Uterine Artery Pulsatility Index; PlGF: placental growth factor; PAPP-A: pregnancy-associated plasma protein A; FMF: Fetal Medicine Foundation; SPR: screen-positive rate; DR: detection rate; BMI: body mass index; Hb: hemoglobin; HL: Hosmer–Lemeshow; RF: Random Forest; LR: logistic regression; DNN: deep neural network; SVM: support vector machine; DT: decision tree; EHR: electronic health record; CART: classification and regression tree; LASSO: least absolute shrinkage and selection operator; XGBoost: extreme gradient boosting; FPG: fasting plasma glucose; HbA1c: hemoglobin A1c; IgA: immunoglobulin A; TPOAb: thyroid peroxidase antibody; t-SNE: t-distributed stochastic neighbor embedding; DCA: decision curve analysis

Author, year	Input features	Validation method	Sensitivity (%)	Specificity (%)	Accuracy (%)	AUC (95% CI)	Other metrics
Yang et al. (2025) [11]	PIERS-ML & fullPIERS	NR	NR	NR	NR	NR	PIERS-ML: AUC-PRC peaked at 0.65 on day 0, declined thereafter; fullPIERS: AUC-PRC 0.2-0.4
Gil et al. (2024) [12]	Maternal risk factors, MAP, UtA-PI, PlGF (PAPP-A not beneficial)	External validation (Spain PREVAL cohort, n = 10,110); fully connected neural network; adjusted for PlGF analyzer; compared with FMF model at 10% SPR	Early PE: 84.4%; Preterm PE: 77.8%; All PE: 55.7%	~90 (fixed 10% SPR)	NR	Early PE: 0.920 (0.864-0.975); Preterm PE: 0.913 (0.882-0.944); All PE: 0.846 (0.820-0.872)	Detection rate (DR) at 10% SPR is comparable to FMF model
Hu et al. (2023) [13]	20 predictors (selected from 33 variables, including maternal clinical and demographic factors)	Training (Aug-Nov 2019, n = 735)/testing (Aug 2020, n = 190)	NR	NR	87.5%	0.946	Good calibration (HL test, calibration plots), decision curve analysis showed clinical net benefit
Li et al. (2023) [14]	Age, BMI, hemoglobin, triglycerides, serum ferritin, fasting blood glucose (1st trimester)	Internal validation (70:30 split; training vs. validation groups)	NR	NR	NR	Training: 0.920; validation: 0.753	Risk formula derived from logistic regression nomogram
Lin and Fang (2023) [15]	Prepregnancy BMI, history of GDM/macrosomia, hypertension, Hb, triglycerides, family history of diabetes, serum ferritin, fasting blood glucose	Train-test split (70:30)	74.1-84.8	87.6-91.4	NR	0.883 (0.846-0.921)-0.950 (0.927-0.973)	Random Forest outperformed logistic regression; Delong test confirmed superiority
Liu et al. (2022) [16]	Maternal characteristics, medical history, prenatal laboratory results, ultrasound results (18 variables)	Cross-validation	42 (95% CI: 41-44)	NR	74 (95% CI: 74-75)	0.86 (95% CI: 0.80-0.92)	Precision: 82 (95% CI: 79-84); Brier score: 0.17 (95% CI: 0.17-0.17)
Liao et al. (2022) [17]	Clinical data from EHR (levels 1-4: preconception, prediagnosis, at GDM diagnosis, 1 week postdiagnosis); simplified model with timing of diagnosis, fasting glucose, and fasting glycemic control	Tenfold cross-validation (2007-2016 discovery set) and temporal validation (2017 cohort)	NR	NR	NR	Super Learner: 0.934 (95% CI: 0.931-0.936) discovery/0.815 (95% CI: 0.800-0.829) validation; Logistic regression: 0.825 (95% CI: 0.820-0.830) discovery/0.798 (95% CI: 0.783-0.813) validation	Ensemble ML (Super Learner: CART, LASSO, RF, XGBoost) outperformed simpler logistic regression but both showed high predictability
Kang et al. (2021) [18]	Age, prepregnancy BMI, FPG, HbA1c, IgA, triglyceride, % of B lymphocytes, progesterone, TPOAb	Fivefold cross-validation	NR	NR	NR	0.772 (0.602-0.942)	Calibration curves showed acceptable agreement; DCA showed good net benefits
Guo et al. (2020) [19]	Age; prepregnancy BMI; first-trimester FPG; diabetes in first-degree relatives	Development: retrospective cohort (n = 3,956) using multiple logistic regression. Model refinement with t-SNE. Internal validation: bootstrap resampling. External/prospective validation: prospective cohort (n = 6,572, 2016, same institution)	NR	NR	NR	Development: 0.69 (95% CI 0.67-0.72). Validation: 0.70 (95% CI 0.68–0.72)	Calibration: reported as “good” (calibration plots well calibrated). t-SNE visualization: distinct separation between GDM and non-GDM regions. Decision curve analysis: positive net benefit for thresholds between 0.05 and 0.78

For PE prediction, the models demonstrated high predictive potential. Gil et al. reported outstanding performance in their external validation of a neural network model, with AUCs of 0.920 for early-onset PE (<34 weeks) and 0.913 for preterm PE (<37 weeks) at a 10% screen-positive rate, which was comparable to the established FMF competing-risks model [12]. Conversely, Yang et al.'s prospective multicountry study, which focused on predicting adverse maternal outcomes after PE diagnosis rather than initial detection, reported a peak AUC for the precision-recall curve (AUC-PRC) of only 0.65 for the PIERS-ML model on the day of admission, which declined over the following two weeks [11]. Liu et al. developed a model for PE diagnosis itself, achieving an AUC of 0.86 [16].

For GDM prediction, the models also showed strong discriminatory power. Hu et al.'s XGBoost model achieved one of the highest AUCs at 0.946, alongside an accuracy of 87.5% [13]. Similarly, Liao et al.'s Super Learner ensemble model for predicting GDM treatment modality showed excellent performance in the discovery cohort (AUC 0.934), though it experienced an expected decrease in the temporal validation cohort (AUC 0.815) [17]. Other studies utilizing nomograms and logistic regression models reported AUCs ranging from 0.69 to 0.92 [14,15,18,19]. Lin and Fang found that their Random Forest model (AUC 0.950) significantly outperformed a logistic regression model (AUC 0.883) for GDM prediction [15].

Beyond AUC, other metrics were variably reported. Sensitivity for PE detection in the first trimester was high in the study by Gil et al. (84.4% for early PE) [12], while Liu et al. reported a sensitivity of 42% for a general PE prediction model [16]. Several studies for GDM conducted rigorous validation and additional analyses. Hu et al. [13], Kang et al. [18], and Guo et al. [19] all reported good model calibration using Hosmer-Lemeshow tests or calibration plots. Furthermore, Hu et al. [13], Kang et al. [18], and Guo et al. [19] performed decision curve analysis (DCA), consistently demonstrating that their models provided a positive net benefit across a range of clinically relevant probability thresholds, indicating potential clinical utility.

The included studies demonstrate that AI and ML models can achieve high predictive accuracy for both PE and GDM, with AUC values frequently exceeding 0.85. However, performance varies based on the specific clinical question (e.g., initial diagnosis vs. outcome prediction), the timing of prediction, the model architecture, and the validation method employed.

PROBAST Risk of Bias Results

The methodological quality of the included studies, as assessed by the PROBAST, revealed that the majority were judged to have a low risk of bias. Specifically, the studies by Yang et al. [11], Gil et al. [12], Hu et al. [13], Liu et al. [16], Liao et al. [17], Kang et al. [18], and Guo et al. [19] all demonstrated a low risk of bias across all domains, including participants, predictors, outcome, and analysis. In contrast, two studies were assessed as having a high overall risk of bias. The study by Li et al. [14] was judged with a high risk of bias in the participants and analysis domains, and the study by Lin and Fang [15] was similarly judged with a high risk of bias in the participants and analysis domains (Table 5).

**Table 5:** Risk of bias assessment for included studies using PROBAST PROBAST: Prediction model Risk of Bias Assessment Tool

Author, year	Participants	Predictors	Outcome	Analysis	Overall risk of bias
Yang et al. (2025) [11]	Low	Low	Low	Low	Low
Gil et al. (2024) [12]	Low	Low	Low	Low	Low
Hu et al. (2023) [13]	Low	Low	Low	Low	Low
Li et al. (2023) [14]	High	Low	Low	High	High
Lin and Fang (2023) [15]	High	Low	Low	High	High
Liu et al. (2022) [16]	Low	Low	Low	Low	Low
Liao et al. (2022) [17]	Low	Low	Low	Low	Low
Kang et al. (2021) [18]	Low	Low	Low	Low	Low
Guo et al. (2020) [19]	Low	Low	Low	Low	Low

Discussion

This systematic review evaluated the current evidence on the diagnostic performance of AI models for the early detection of PE and GDM. The analysis of nine included studies reveals a rapidly evolving field where ML and AI demonstrate considerable promise, achieving high predictive accuracy with AUC values frequently exceeding 0.85. The findings, however, are nuanced and must be interpreted within the context of significant heterogeneity in clinical objectives, model architectures, and crucially, methodological rigor. The superior performance of models like the neural network validated by Gil et al. [12] for first-trimester PE prediction (AUC 0.920-0.913) and the XGBoost model by Hu et al. [13] for GDM (AUC 0.946) suggests that AI can potentially outperform traditional statistical methods and even compete with established clinical benchmarks like the FMF model. This aligns with a growing body of literature across medicine that posits AI's strength in identifying complex, nonlinear interactions within high-dimensional clinical data that might elude conventional approaches. However, the concurrent observation of more modest performance in other studies, such as the declining AUC-PRC for adverse outcomes in the PIERS-ML model [11] and the significant drop in performance from training to validation in the nomogram by Li et al. [14] (AUC 0.920 to 0.753), serves as a critical reminder that reported performance is intensely context-dependent. These discrepancies are not merely academic; they underscore the fundamental challenge in translating a well-performing algorithm into a reliable clinical tool.

The distinction in predictive purpose among the PE studies is particularly illustrative of this context dependence. The model by Gil et al. [12] aimed for the initial detection of PE, a screening task, and achieved high performance by leveraging first-trimester biomarkers like PlGF and UtA-PI, which are known to be strongly associated with placental dysfunction years before clinical onset, reflecting underlying angiogenic pathophysiology. In contrast, the study by Yang et al. [11] addressed a different, though equally critical, clinical question: risk stratification and outcome prediction in women already diagnosed with PE. Their finding that predictive power for adverse outcomes was highest at admission (AUC-PRC 0.65) and waned over time is clinically intuitive; as management is initiated, iatrogenic and therapeutic interventions alter the natural history of the disease, making subsequent outcomes harder to predict from admission data alone. This also reflects the use of different clinical and temporal predictors compared with screening models. This does not invalidate the model but rather clarifies its utility window. Similarly, Liu et al. [16] focused on diagnosis itself, achieving a robust AUC of 0.86. This trichotomy, screening, diagnosis, and prognostication, is often conflated in AI literature but represents distinct clinical workflows with different data availability, time constraints, biomarker sets, and consequences of error. A model effective for one purpose may be entirely unsuitable for another, a nuance that must be forefront in both development and implementation strategies.

For GDM, the models demonstrated a consistent ability to identify high-risk women, though the optimal feature set and model architecture remain areas of active investigation. The high performance of ensemble methods like XGBoost [13] and the Super Learner [17] suggests that combining multiple weak predictors through advanced algorithms can capture the multifactorial etiology of GDM more effectively than single models. However, interpretability and generalizability of these ensemble models remain important barriers, which must be addressed to avoid premature clinical adoption. Liao et al.'s [17] work is especially noteworthy for its ambitious aim of predicting not just GDM diagnosis, but the required treatment modality, demonstrating the potential for AI to guide personalized care pathways. Their observed performance decrease from discovery (AUC 0.934) to temporal validation (AUC 0.815) is a classic and valuable demonstration of "AI drift" and the attenuation of performance when models are applied to data from a different time period, a crucial test of real-world viability. The studies relying on logistic regression to create nomograms [14,15,18,19] reported a wider range of AUCs (0.69 to 0.92). While often less complex, these models offer the advantage of interpretability, a key factor for clinical adoption. The finding by Lin and Fang [15] that their Random Forest model significantly outperformed logistic regression (AUC 0.950 vs. 0.883) is a powerful argument for the use of nonlinear ML methods, provided the loss of interpretability can be mitigated through explainable AI (XAI) techniques.

Beyond discrimination (AUC), the move toward more comprehensive model validation is a positive trend in the field. The reporting of calibration metrics and DCA in several studies [13,18,19] represents a significant advancement over reviews conducted just a few years ago. A model can have excellent AUC yet be poorly calibrated, meaning its predicted probabilities do not match the observed frequencies; this would render it dangerous for clinical decision-making, where probability thresholds guide actions. The good calibration reported by Hu, Kang, and Guo et al. is therefore as important as their high AUC values. Furthermore, DCA moves beyond statistical metrics to answer a pragmatic clinical question: across a range of risk thresholds, does using this model to guide decisions (e.g., to test or treat) provide a better net benefit than alternative strategies (treat all or treat none)? The demonstration of a positive net benefit in the few studies reporting DCA [13,18,19], though all retrospective, suggests potential clinical utility of these tools, while acknowledging that external validation in diverse populations remains limited.

The PROBAST assessment provides an essential lens through which to interpret these performance results. The fact that seven studies were judged as low risk of bias [11-13,16-19] is encouraging and indicates a growing awareness of methodological best practices, particularly the importance of prospective design [11,12,18] and external or temporal validation [12,17,19]. External validation, as performed by Gil et al. [12] in a Spanish cohort with a UK-developed model and by Guo et al. [19] with a prospective cohort, is the gold standard for assessing generalizability and provides the most credible evidence of a model's readiness for broader use. Conversely, the high risk of bias identified in two studies [14,15] highlights persistent pitfalls. The use of case-control [13] or selectively sampled retrospective designs [14,15] inflates performance estimates because the model is trained and tested on a population that does not represent the true clinical screening scenario, where the prevalence of disease is lower and the spectrum of presentation is wider. Furthermore, reliance on simple data splitting or cross-validation without external validation [14,15,18] almost guarantees optimistic performance due to overfitting to the idiosyncrasies of a single dataset. These methodological shortcomings are not unique to this review but are endemic in the broader AI in medicine literature, often leading to a "jingle fallacy" where models with similar AUCs are deemed equivalent without scrutiny of the evidence strength behind each estimate. Other common biases include small sample sizes, incomplete handling of missing data, feature selection bias, and lack of model reproducibility, all of which can undermine the reliability and generalizability of AI models.

When placed in the context of existing literature, our findings both confirm and extend previous knowledge, while acknowledging the possibility of publication bias, as studies with poor performance are less likely to be published, potentially inflating the apparent promise of AI. The high performance for first-trimester PE prediction is consistent with systematic reviews by Townsend et al. [20] and Beam et al. [21], who also found that models incorporating angiogenic biomarkers like PlGF achieve superior performance compared to those using only maternal history. Our review adds to this by including the recent work of Gil et al. [12], which directly benchmarks an AI model against the clinical gold standard (FMF model), a comparison that is still rare. For GDM, our results corroborate the review by Artzi et al. [22], which found that ML models generally outperform those based on traditional regression. However, we observed a wider range of performance, which we attribute to a more critical assessment of study design and bias. A key contribution of this review is the inclusion of studies like Liao et al. [17] that push beyond mere diagnosis toward predicting management needs, a crucial next step for clinical impact that was identified as a gap by Liu et al. [23]. Furthermore, the emphasis on calibration and clinical utility via DCA addresses a specific criticism levied by Collins and Moons [24] against the AI field's overreliance on discrimination metrics alone.

Limitations

This systematic review has several limitations. First, the number of included studies is relatively small (n = 9), and the field is characterized by significant clinical and methodological heterogeneity, precluding a formal meta-analysis to derive pooled estimates of performance. Second, our search was limited to major databases and English-language publications, potentially omitting relevant studies published in other languages or in grey literature. Third, the risk of bias assessment, while conducted using a robust tool, was reliant on the information reported in the publications; incomplete reporting, particularly regarding participant flow and handling of missing data, may have led to an unclear or potentially optimistic bias assessment for some domains. Fourth, the review could not deeply evaluate the computational reproducibility of the models, as access to code and algorithms was not available for any of the studies. Finally, the field is advancing at a rapid pace, and new studies with potentially superior methodologies or models are continuously emerging, meaning this review provides a snapshot of the evidence at a specific point in time.

## Conclusions

AI models show high potential for the early detection of PE and GDM, with several studies achieving excellent discriminatory performance and demonstrating promising clinical utility. The most robust evidence comes from prospectively designed or externally validated studies that utilize advanced ML techniques like neural networks and ensemble methods, incorporate key biomarkers, and move beyond simple discrimination to assess calibration and net benefit. However, the field remains hampered by a subset of studies with high risk of bias, often due to case-control designs and a lack of external validation, which threatens the validity and generalizability of their findings. Future research must prioritize prospective, randomized trials embedded in clinical workflows to evaluate the actual impact of these AI tools on patient outcomes, resource utilization, and health equity. Developers should adhere to TRIPOD+AI reporting guidelines to enhance transparency and reproducibility. While the promise of AI in transforming prenatal care is undeniable, realizing this promise requires a steadfast commitment to methodological rigor, comprehensive validation, and ultimately, proof of clinical effectiveness.

## Appendices

**Table 6:** Search strategy for the rest of databases

Database	Search string
Scopus	(TITLE-ABS-KEY("preeclampsia" OR "pre-eclampsia" OR "gestational diabetes" OR "GDM") AND TITLE-ABS-KEY("artificial intelligence" OR "machine learning" OR "deep learning" OR "predictive model*" OR "risk stratification" OR "screening") AND TITLE-ABS-KEY("diagnosis" OR "diagnostic performance" OR "sensitivity" OR "specificity" OR "accuracy" OR "AUC")))
EMBASE	('preeclampsia'/exp OR 'pre-eclampsia' OR 'gestational diabetes mellitus'/exp OR 'GDM') AND ('artificial intelligence'/exp OR 'machine learning'/exp OR 'deep learning' OR 'predictive model*' OR 'risk stratification' OR 'screening') AND ('diagnosis'/exp OR 'diagnostic performance' OR 'sensitivity' OR 'specificity' OR 'accuracy' OR 'AUC')
IEEE Xplore	("preeclampsia" OR "pre-eclampsia" OR "gestational diabetes" OR "GDM") AND ("artificial intelligence" OR "machine learning" OR "deep learning" OR "predictive model*" OR "risk stratification" OR "screening") AND ("diagnosis" OR "diagnostic performance" OR "sensitivity" OR "specificity" OR "accuracy" OR "AUC")
ACM Digital Library	("preeclampsia" OR "pre-eclampsia" OR "gestational diabetes" OR "GDM") AND ("artificial intelligence" OR "machine learning" OR "deep learning" OR "predictive model*" OR "risk stratification" OR "screening") AND ("diagnosis" OR "diagnostic performance" OR "sensitivity" OR "specificity" OR "accuracy" OR "AUC")

## References

[1] Preda A, Pădureanu V, Moța M (2021). Analysis of maternal and neonatal complications in a group of patients with gestational diabetes mellitus. Medicina (Kaunas).

[2] Chang KJ, Seow KM, Chen KH (2023). Preeclampsia: recent advances in predicting, preventing, and managing the maternal and fetal life-threatening condition. Int J Environ Res Public Health.

[3] Yang Y, Wu N (2022). Gestational diabetes mellitus and preeclampsia: correlation and influencing factors. Front Cardiovasc Med.

[4] Oladipo AF, Jayade M (2024). Review of laboratory testing and biomarker screening for preeclampsia. BioMed.

[5] Lorenzo-Almorós A, Hang T, Peiró C, Soriano-Guillén L, Egido J, Tuñón J, Lorenzo Ó (2019). Predictive and diagnostic biomarkers for gestational diabetes and its associated metabolic and cardiovascular diseases. Cardiovasc Diabetol.

[6] Davidson L, Boland MR (2021). Towards deep phenotyping pregnancy: a systematic review on artificial intelligence and machine learning methods to improve pregnancy outcomes. Brief Bioinform.

[7] Medjedovic E, Stanojevic M, Jonuzovic-Prosic S, Ribic E, Begic Z, Cerovac A, Badnjevic A (2024). Artificial intelligence as a new answer to old challenges in maternal-fetal medicine and obstetrics. Technol Health Care.

[8] Giaxi P, Vivilaki V, Sarella A, Harizopoulou V, Gourounti K (2025). Artificial intelligence and machine learning: an updated systematic review of their role in obstetrics and midwifery. Cureus.

[9] Malik S, Ishaq S, Farzooq H, Joya HU (2025). Artificial intelligence in predicting pregnancy complications: a systematic review and meta-analysis of preeclampsia and gestational diabetes mellitus. Indus J Biosci Res.

[10] Wolff RF, Moons KG, Riley RD (2019). PROBAST: a tool to assess the risk of bias and applicability of prediction model studies. Ann Intern Med.

[11] Yang G, Montgomery-Csobán T, Ganzevoort W (2025). Consecutive prediction of adverse maternal outcomes of preeclampsia, using the PIERS-ML and fullPIERS models: a multicountry prospective observational study. PLoS Med.

[12] Gil MM, Cuenca-Gómez D, Rolle V (2024). Validation of machine-learning model for first-trimester prediction of pre-eclampsia using cohort from PREVAL study. Ultrasound Obstet Gynecol.

[13] Hu X, Hu X, Yu Y, Wang J (2023). Prediction model for gestational diabetes mellitus using the XG Boost machine learning algorithm. Front Endocrinol (Lausanne).

[14] Li R, Yuan K, Yu X, Jiang Y, Liu P, Zhang K (2023). Construction and validation of risk prediction model for gestational diabetes based on a nomogram. Am J Transl Res.

[15] Lin Q, Fang ZJ (2023). Establishment and evaluation of a risk prediction model for gestational diabetes mellitus. World J Diabetes.

[16] Liu M, Yang X, Chen G (2022). Development of a prediction model on preeclampsia using machine learning-based method: a retrospective cohort study in China. Front Physiol.

[17] Liao LD, Ferrara A, Greenberg MB (2022). Development and validation of prediction models for gestational diabetes treatment modality using supervised machine learning: a population-based cohort study. BMC Med.

[18] Kang M, Zhang H, Zhang J (2021). A novel nomogram for predicting gestational diabetes mellitus during early pregnancy. Front Endocrinol (Lausanne).

[19] Guo F, Yang S, Zhang Y, Yang X, Zhang C, Fan J (2020). Nomogram for prediction of gestational diabetes mellitus in urban, Chinese, pregnant women. BMC Pregnancy Childbirth.

[20] Townsend R, Khalil A, Premakumar Y (2019). Prediction of pre-eclampsia: review of reviews. Ultrasound Obstet Gynecol.

[21] Beam K, Sharma P, Levy P, Beam AL (2024). Artificial intelligence in the neonatal intensive care unit: the time is now. J Perinatol.

[22] Artzi NS, Shilo S, Hadar E (2020). Prediction of gestational diabetes based on nationwide electronic health records. Nat Med.

[23] Liu Q, Zhu S, Zhao M (2025). Machine learning approaches for predicting fetal macrosomia at different stages of pregnancy: a retrospective study in China. BMC Pregnancy Childbirth.

[24] Collins GS, Reitsma JB, Altman DG, Moons KG (2015). Transparent reporting of a multivariable prediction model for individual prognosis or diagnosis (TRIPOD): the TRIPOD statement. Br J Surg.

